# Wastewater-based surveillance for community exposome surveillance, Louisville, Kentucky

**DOI:** 10.1007/s43832-026-00376-5

**Published:** 2026-03-12

**Authors:** Lu Cai, Rochelle H. Holm, Donald J. Biddle, Charlie H. Zhang, Daymond Talley, Ted Smith, J. Christopher States

**Affiliations:** 1 Department of Pediatrics, Pediatrics Research Institute, University of Louisville, Louisville, KY USA; 2 Center for Integrative Environmental Health Sciences, University of Louisville, 505 S. Hancock St., Louisville, KY 40202 USA; 3 Center for Healthy Air Water and Soil, Christina Lee Brown Envirome Institute, University of Louisville, 302 E. Muhammad Ali Blvd., Louisville, KY 40202 USA; 4 Department of Geographic and Environmental Sciences, University of Louisville, Louisville, KY USA; 5 Center for Geographic Information Sciences, University of Louisville, Louisville, KY USA; 6 Louisville/Jefferson County Metropolitan Sewer District, Morris Forman Water Quality Treatment Center, Louisville, KY USA; 7 Department of Pharmacology and Toxicology, University of Louisville, 505 S. Hancock St., Louisville, KY 40202 USA

**Keywords:** Exposome, Heavy metals, Neighborhood, Pollution, Sewer, Wastewater-based epidemiology

## Abstract

Exposomics research has primarily focused on individual exposures relevant to precision environmental health. Community exposomics is an emerging concept that seeks to connect community-level exposures to public health, but clear, practical ways to scale surveillance from individuals to communities—especially using wastewater to determine community chemical exposures—remain limited. We address this issue by applying wastewater-based surveillance for community exposome surveillance, allowing detection of neighborhood- and multi-neighborhood-scale exposure to environmental toxicants. Influent wastewater samples from 24 sites and tap water samples from five sites in the same areas were analyzed using an Inductively Coupled Plasma Quadrupole Mass Spectrometer to quantify 26 metals. Exposure levels vary across the county: 20 of 24 sites had at least one metal level exceeding 1 standard deviation (SD) above the mean and were designated areas of concern. Additionally, 14 of the 20 sites were designated as having increased concern, with levels greater than 2 SD above the mean. Our findings contribute to a better understanding of how wastewater-based surveillance can serve as a tool for transitioning from individual- to community-scale exposome assessment, with neighborhood- and multi-neighborhood-scale metal variance observed across a single county. A place-based community health exposomics surveillance model that leverages wastewater may be a promising approach for future public health action.

**Supplementary Information:**

The online version contains supplementary material available at 10.1007/s43832-026-00376-5.

## Introduction

Chronic exposure to environmental pollutants is increasingly recognized as a major contributor to common chronic diseases, often outweighing individual genetic predisposition [1–6]. Exposomics research aims to measure the totality of exposures across the life course, from conception to death [5, 6]. Thus far, exposomics has focused on individual exposures in the context of precision environmental health [5]. A new concept of community exposomics is emerging in public health, moving from reliance on individuals seeking healthcare services to a more place-based community health surveillance model. Wastewater surveillance may be used as a place-based, continuous tool to aid in the surveillance of health outcomes of chemical exposures [7]. Currently, community exposure measurements commonly include monitoring air pollution, air temperature, and drinking water [8]. Existing community exposure measurements may provide a more complete picture of community-level exposomics by adding neighborhood- and multi-neighborhood-scale wastewater surveillance.

A detailed framework for scaling from individuals to community-scale surveillance—leveraging the additive value of wastewater to determine community chemical exposures—has yet to be developed. Wastewater has been used to surveil toxicants, including illicit drugs [9–11], tobacco metabolites [10], and biological agents as weapons [12]. Xu et al. [13] estimated the consumption of tobacco through the analysis of metal concentrations in wastewater. In contrast, for pathogen health threats, the range of wastewater-based epidemiology (WBE) geographic scales is well established, spanning buildings and neighborhoods to entire cities [14–18]. Several metals are International Agency for Research on Cancer Group I carcinogens (e.g., As, Be, Cr, Cd, and Ni) [19]; they are linked to a variety of diseases, including cancers [20–21], cardiovascular disease [1, 2], diabetes [22–24], and neurological diseases [25–27]. While Markosian and Mirzoyan [28] proposed extending WBE as a novel approach to assess population-level exposure to metals, wastewater surveillance at neighborhood and multi-neighborhood scales—as part of comprehensive, community-wide exposome surveillance—has not yet been studied.

Metals can enter the wastewater system through piped system discharge, environmental factors (e.g., wind, rain, or surface runoff) in areas where sewer and stormwater pipes are combined, and/or via human excreta resulting from ingestion and inhalation. Human metal excretion concentrations vary widely among individuals [29]. Metals in drinking water can enter the wastewater system through two primary pathways: (1) metals ingested in drinking may be excreted by humans; and (2) constituents such as lead (Pb) can leach from water service lines and residential plumbing and fixtures—installed before the passage of the Safe Drinking Water Act Amendments of 1986 [30]—and enter the sewer via non- ingestion activities (e.g., grey water discharge). Any wastewater surveillance would thus need to be paired with drinking water surveillance to provide a complementary, localized, measure of human exposure for regional public health concerns associated with metals exposure.

Wastewater surveillance is a community health tool that may produce data to inform public health interventions and policy decisions [31]. Approximately 96% of urban households in the U.S. have their human excreta enter a piped sewer connection [32], where exposomics wastewater surveillance may be feasible. Herein, we selected Jefferson County, Kentucky, as a study site to apply wastewater-based metals surveillance as a tool for community exposome surveillance to detect smaller neighborhood- and multi-neighborhood-scale elevations in human exposure to environmental toxicants.

## Methods

### Wastewater study sites

**Fig. 1:**
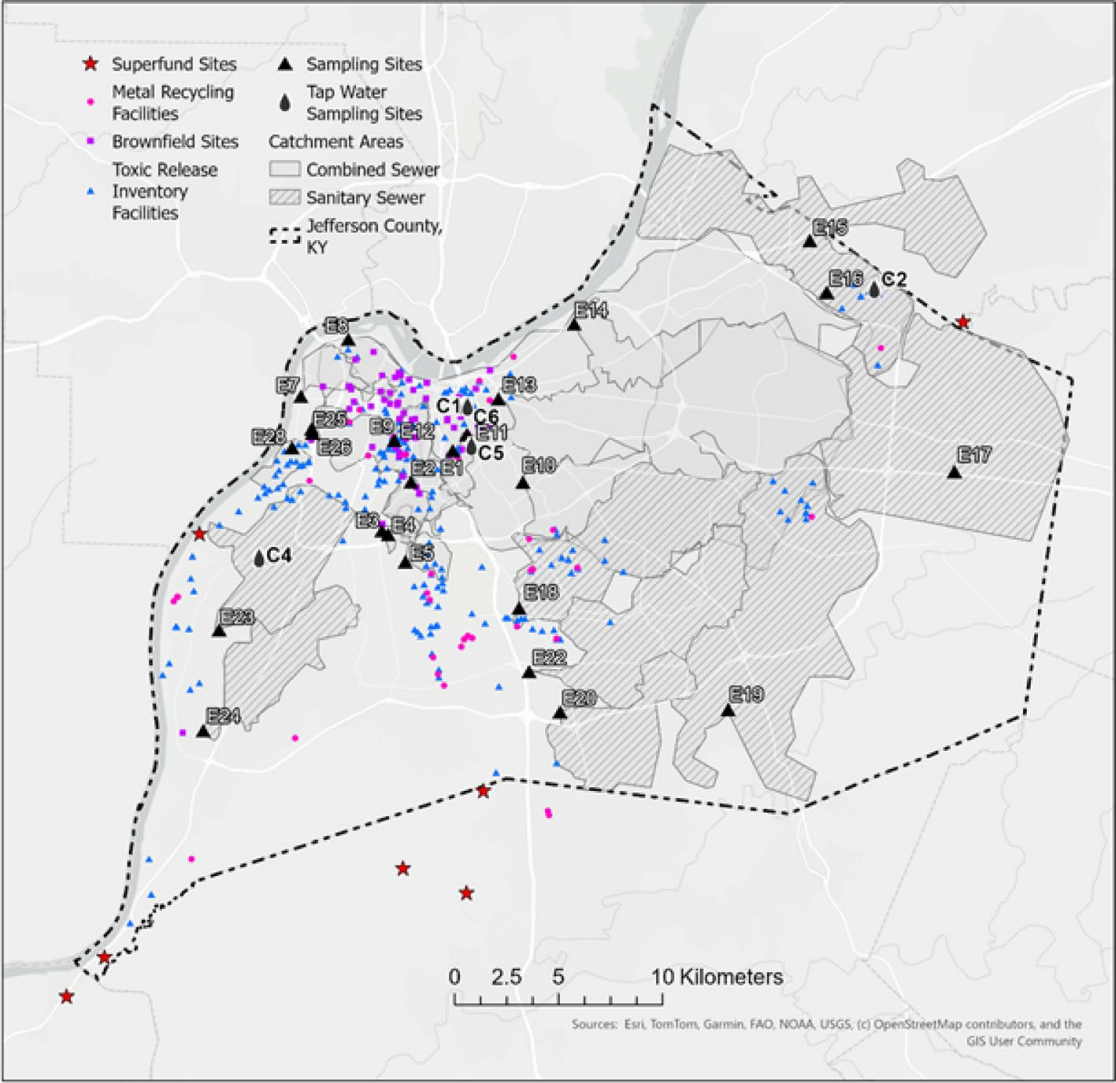
Location of wastewater and tap water sampling sites in Jefferson County, Kentucky, USA, bordered on the north and west by the Ohio River. Wastewater samples were collected from 24 locations (E1–E28). Table 1 summarizes whether each sewershed included combined sewer overflow and describes key characteristics. Tap water was sampled at five sites (C1, C2, C4, C5, and C6). Sanitary sewersheds, Superfund sites, metal recycling facilities, Brownfield sites, and Toxic Release Inventory sites are indicated

Our study took place in Jefferson County, Kentucky (USA) (Fig. 1). All samples were collected prior to wastewater treatment. Twenty-four sewershed locations were selected across the county to represent sub-county geographic and demographic variability, with purposive bias toward sites with known environmental exposures and risks, including near a Superfund site, Brownfield sites, metal recycling facilities, and Toxics Release Inventory sites. In addition, sites were selected to ensure fair countywide geographic distribution, although this purposive design may have missed areas with low metal loads. Environmental exposure location data were obtained from the United States Environmental Protection Agency (EPA) Facility Registry Service (FRS) databases [33]. Several sampling locations involved combined sewer and stormwater pipes (*N* = 15), which may dilute wastewater samples or introduce toxicants from the environment via surface runoff.

**Table 1:** Wastewater sample site characteristics, Jefferson County, Kentucky (USA)

Sampling site^a^	Household income (USD) 2020 Median (ACS 5 Yr)	Population	Race: White (%)	Race: Black (%)	Hispanic Population (%)	Area (km^2^)	Does sewershed include combined sewer overflow?	Material and construction year
E1	0	0	0	0	0	0.01	Yes	Clay 1916
E2	34,490	8258	51	32	10	4	Yes	Clay 1916
E3	36,812	3459	57	24	13	1	Yes	Concrete 1928
E4	37,059	1610	61	22	10	1	Yes	Concrete 1938
E5	40,168	3587	56	21	12	3	Yes	Concrete 1930
E7	31,050	10,949	13	82	2	5	Yes	Concrete 1924
E8	27,752	9073	57	36	2	5	Yes	Concrete 1950
E9/E12^*b*^	27,517	23,751	55	32	6	12	Yes	Brick 1910
E10	65,791	145,346	70	14	7	112	Yes	Concrete 1932
E11	56,672	8838	80	9	3	3	Yes	Brick 1912
E13	74,500	95,603	80	7	5	80	Yes	Concrete 1977
E14	101,140	11,444	91	2	3	12	Yes	PVC 1993
E15	86,478	40,824	73	11	6	67	–	(Treatment plant)
E16	81,994	5781	70	12	5	11	No	Concrete 1969
E17	108,021	37,193	78	8	4	88	–	(Treatment plant)
E18	55,433	78,206	52	27	13	55	No	Concrete 1954
E19	76,796	60,885	75	12	7	80	–	(Treatment plant)
E20	72,401	24,969	70	15	9	23	No	Concrete 1995
E22	61,923	45,148	62	19	12	37	No	Concrete 1994
E23	45,794	37,972	47	41	7	28	No	Concrete 1978
E24	53,857	23,135	71	17	6	21	No	Concrete 1979
E25	61,081	309,184	71	16	5	242	Yes	Concrete 1958
E26	28,054	41,777	38	52	5	20	Yes	Concrete 1912
E28	20,000	74	11	80	4	3	Yes	Concrete 1960
^a^ Population variables within each sewershed area were obtained from the U.S Census Bureau using the Enrich tool in ArcGIS Pro. ^b^ One site (E9/E12) was sampled at two separate times on the same day to evaluate for site variability, approximately one hour apart with two separate field sample collection technicians.								

### Wastewater sample collection and handling

Influent grab wastewater samples were initially collected on December 12, 2022 (*N* = 25). Each sample was collected into four 50 ml polypropylene tubes per site and transported on ice to the University of Louisville for analysis and/or storage. One field rinse blank was prepared using per- and polyfluoroalkyl substance free water poured over the sampling equipment to evaluate quality assurance and potential metal contamination during collection. Additionally, one site was sampled twice on the same day (E9 and E12) as a field replicate to assess site variability.

An additional set of influent 24-hour composite wastewater samples was collected at Site E23 from January 9 to 25, 2024 (*N* = 9) on each of three consecutive days for three consecutive weeks to evaluate intra-site variability.

### Tap water study sites

Five tap water locations (sites C1, C2, C4, C5, and C6) were selected across the county to represent geographic and demographic sub-county variability, with purposive site selection aligned with the wastewater study sites. In the study area, there are two sources from the water district supplied by Louisville Water Company [34]: the Ohio River and an adjacent aquifer. Water from both sources was tested to ensure compliance with regulatory limits.

### Tap water sample collection and handling

After a 1–2 min flush to ensure it represented running tap water, samples of tap water were collected into 15 mL metal-free tubes at five residential locations (Fig. 1). Samples were transported to the University of Louisville for analysis.

### Metal analysis

Wastewater and tap water samples were stored at  − 20 °C until analysis. Prior to analysis, the samples were thawed and thoroughly mixed. Triplicate 1 mL aliquots were transferred to 15 mL metal-free tubes (VWR #89049-172), and 3 mL of 70% nitric acid (trace metal grade, Fisher Scientific Cat#A509-P500) was added to each tube, followed by mixing. Samples were digested in a 65 °C shaker for 5 h until the solution became clear with no residues. After digestion, samples were transferred to a fume hood, cooled to room temperature, and 1 mL was diluted 10-fold with deionized water (Millipore, Milli-Q Academic). The resulting assay volume was 10 mL. Metal concentrations were measured using an Agilent 7800 Inductively Coupled Plasma Quadrupole Mass Spectrometer (ICP-MS, Agilent Technologies, Japan). Optimization was performed using a performance check with a 1 ppb tuning solution, and the assay program auto-tuned with a 10 ppb tuning solution (Agilent Cat#: 5188–6564). The autosampler SPS 4 was used for sample introduction. The analysis was performed to test 26 metals (aluminum, antimony, arsenic, barium, beryllium, cadmium, calcium, chromium, cobalt, copper, iron, lead, magnesium, manganese, molybdenum, nickel, platinum, potassium, selenium, silver, sodium, thallium, thorium, uranium, vanadium, and zinc). Calibration standards for 25 metals (25-Element Environmental Calibration/Quality Control Standard; Cat#: IV-STOCK-50; certified reference material in a nitric/hydrofluoric acid matrix; traceable to National Institute of Standards and Technology (NIST)) and a platinum standard solution (Cat#: CGPTN1) were purchased from Inorganic Ventures (Christiansburg, VA). Serial metal standard dilutions were prepared using the same acid matrix as the samples. The internal standard (Cat#: 5188–6525) was purchased from Agilent Technologies and contains seven elements (Li, Sc, Ge, Rh, In, Tb, and Bi) that were analyzed together with the samples. The recovery rate typically ranged from 95 to 105%.

To ensure accuracy and comparability between runs, including samples tested at early and late times of larger batch run (30–150 samples per run), we included all blanks of deionized water, reagents (deionized water plus the solvents used for sample treatment), and a certified Standard Reference Material from NIST (Cat # 1643f) between an average of 25 samples. The assay program was run in Agilent MassHunter software in He mode. Each sample was analyzed in triplicate, and the triplicate measurements were averaged to obtain the final mean value (Table S1). Standard curves were created, and detection limits were established for each metal (Table S1).

### Data analysis

Geographic Information Systems (GIS) data from the Louisville–Jefferson County Metropolitan Sewer District [35], including sewer mains, manhole points, and property service connection features, were queried against parcel data from the Jefferson County Property Valuation Administration [36] in ArcGIS Pro to delineate sewershed areas for each sampling location. Demographic characteristics for each sewershed, including total population, median household income per year, and race demographics, were generated using ArcGIS Pro’s Data Apportionment (Enrich) tools with U.S. Census Bureau datasets. The resulting sewershed data were then compared with household income patterns and overlaid with environmental exposure sources, including Superfund sites, metal recycling facilities, Brownfields, and Toxic Release Inventory sites, derived from publicly available U.S. EPA FRS data [33].

For each of the 26 metals studied, sample concentrations greater than 1 and 2 standard deviations (SD) above the mean were designated as areas of concern and increased concern, respectively, as thresholds for important concentrations.

## Results

### Places of concern

**Fig. 2:**
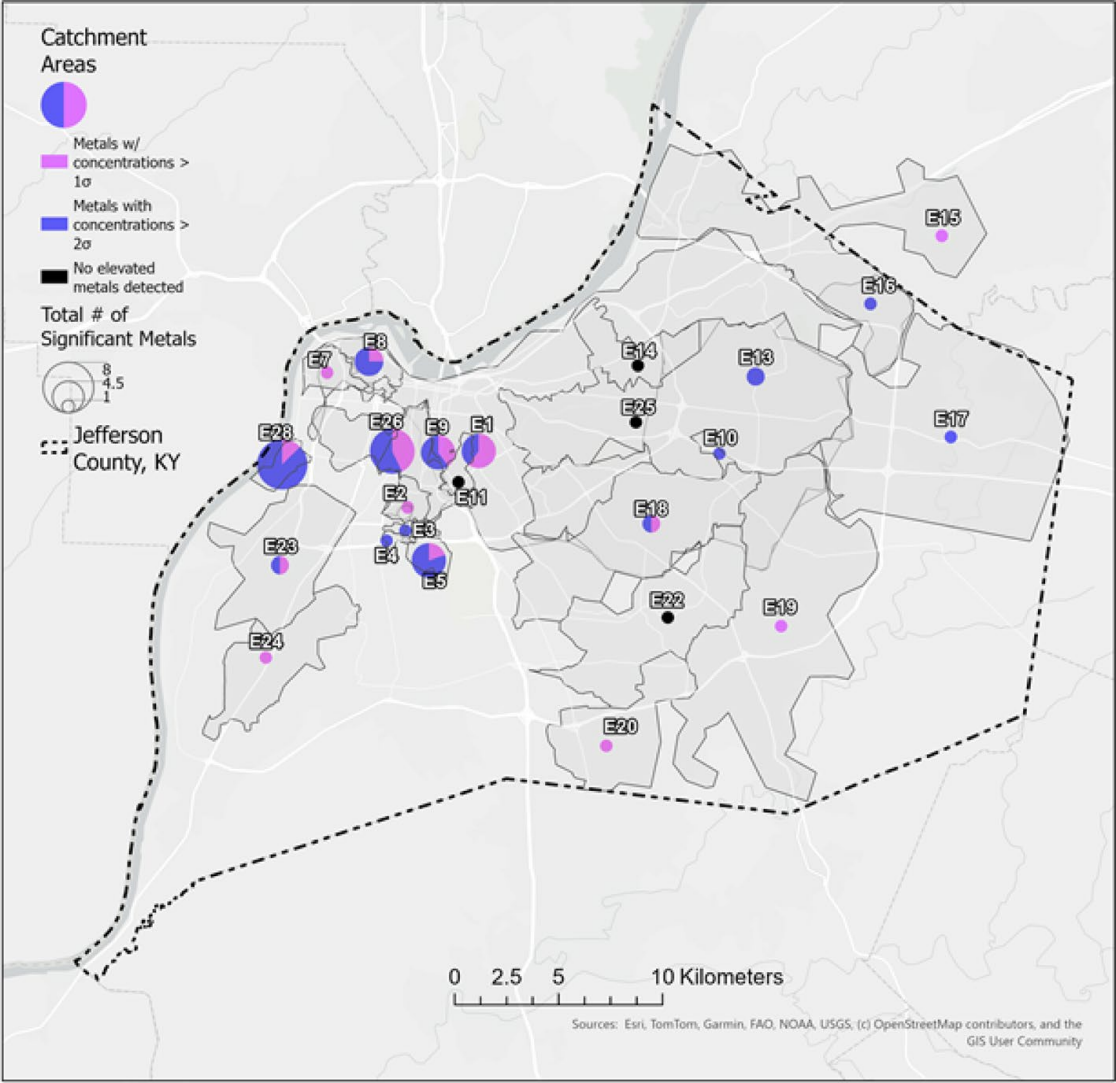
Location of wastewater sewershed areas (*N* = 24) with thresholds of important metal concentrations in Jefferson County, Kentucky, USA. For each sampling location, the pie chart diameter is proportional to the number of metals with concentrations exceeding one standard deviation above the mean. Pie chart indicates the proportion of metals exceeding 1 (pink) and/or 2 (blue) standard deviations above the mean

The wastewater data suggest several metals of concern, with levels of community exposure varying across the county: 20 of the 24 sites had one or more metal levels greater than 1 SD above the mean and were designated areas of concern. Additionally, 14 of the 20 sites were designated as areas of increased concern with levels greater than 2 SD above the mean (Fig. 2; Table S2).

**Fig. 3:**
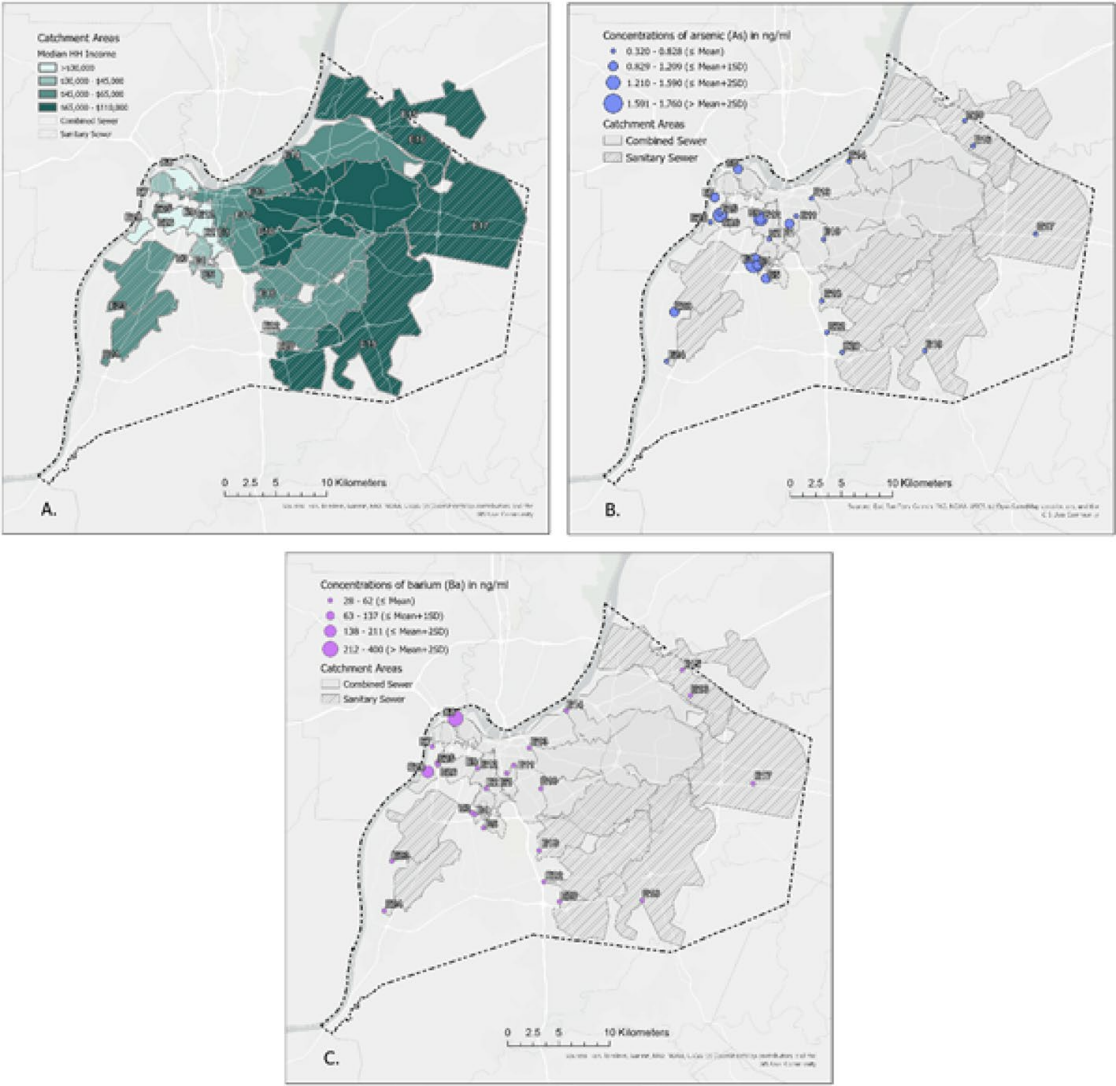
Maps illustrating the spatial relationships between median household income per year (**A**) and arsenic (**B**) and barium (**C**) wastewater concentrations (circles) across 24 sampling sites in Jefferson County, Kentucky (USA). In map A, green shading indicates median household income of each sampled catchment area. In maps B and C, point size is proportional to the arsenic and barium concentrations, respectively

Results show environmental metal exposure is present across a range of socio-economic inclusion. Median household income in areas with metal levels of concern ranged from US$20,000 to US$108,000. Among the two neighborhood sites with the highest count of metals exceeding 1 SD above the mean—Site E26 (seven metals; median household income of US$28,054) and Site E28 (eight metals; median household income of US$20,000)—median household income was low (Table 1; Fig. 3, panel A).

### Proximity to other environmental sources of pollution

In the study area, potential sources of environmental pollutants are Superfund sites, metal recycling facilities, Brownfield sites, and Toxic Release Inventory sites—all of which could have contributed to the observed metal concentrations in wastewater (Fig. 1). Most environmental sources of pollution are concentrated in the northwest portion of Jefferson County, characterized by high proportions of low-income households and African American residents (Fig. 3, Panel A). Using As as an example (Fig. 3, Panel B) of a public health concern, countywide concentrations reached up to 1.76 ng/mL, with the highest concentrations in the northwestern part near potential pollution sources. Concentrations decreased with increasing geographic distance from these sources, particularly in the east and southeastern, to 0.32–0.69 ng/mL. The As mean level in the five countywide tap water samples was 0.51 ng/mL, indicating that drinking water is unlikely to be the source of the observed wastewater concentration. Using Ba as another (Fig. 3, Panel C) metal of public health concern, countywide concentrations reached up to 399 ng/mL, with the highest levels again observed in the northwestern region, closest to potential environmental sources of pollution. Ba concentrations again decreased in the east and southeastern zones to 37–44 ng/mL. The mean Ba level in the five countywide tap water samples was 24 ng/mL, indicating that drinking water is unlikely to be the source of Ba in wastewater.

### Intra-site variability

One site (E9/E12) was sampled with a single grab at two separate times on the same day to evaluate site variability, approximately 1 h apart, with two separate field sample collection technicians. Results indicate variance in concentration, with most metals at levels of concern in common (Table S2). While sample E9 was elevated in Be, Na, Al, K, and Pt, sample E12 was elevated in Be, Na, Al, K, Fe, and As. The sampling site is also uniquely close to a neighborhood heavily contaminated with As, historically associated with pesticide manufacturing and lumber treatment. Much (but not all) of the neighborhood had its topsoil removed and replaced [37]. Whether the variability at this site is due to temporal differences or to historical contamination requires further investigation.

The E23 grab sample had a Pb level of concern (8.8 ng/mL). Therefore, we revisited the site to assess intra-site variability using a different field-collection method. A 24 hour composite sample was collected on 9 separate days over 3 weeks in January 2024 (Table S3). Lead concentration in composite samples from site E23 varied from 1.52 to 11.51 ng/mL (mean = 3.67 ng/mL, median = 2.21 ng/mL; standard deviation = 3.41 ng/mL) over the 3 weeks.

### Contributing tap water

We sampled tap water from five sites within Jefferson County (Table S4) served by the Louisville Water Company. The measured metal concentrations were each below the U.S. EPA [38] maximum contaminant levels or action levels and generally below the levels detected in wastewater samples.

### Comparison to service area wastewater discharge permits

In the absence of metal concentration standards for influent wastewater, our results were also compared with the limits established by service area wastewater discharge permits. The permit limits [39] vary across the county; results were below each standard (Table 2). However, metals in the samples are likely diluted and do not necessarily reflect higher concentrations potentially from industrial discharges.

**Table 2:** Maximum wastewater grab sample concentration of studied metals and permit standards by area

Metal		Permit limits by area^a^				
	Maximum concentration observed (*N* = 25) (ng/mL)	Site E15, E16 (ng/mL)	Site E17 (ng/mL)	Site E19 (ng/mL)	Site E18, E20, E22, E23, E24 (ng/mL)	Site E1, E2, E3, E4, E5, E7, E8, E9, E12, E10, E11, E13, E14, E25, E26, E28 (ng/mL)
Aluminum	20,354.55	–	–	–	–	–
Antimony	1.17	–	–	–	–	–
Arsenic	1.76	30	340	1700	570	170
Barium	399.41	–	–	–	–	–
Beryllium	2.16	–	–	–	–	–
Cadmium	0.48	3	40	550	47	77
Calcium	3,410,126.39	–	–	–	–	–
Chromium	6.88	4470	5000	5000	5000	5000
Cobalt	7.49	–	–	–	–	–
Copper	72.08	430	1400	1400	7900	2410
Iron	1154.79	–	–	–	–	–
Lead	8.80	60	830	830	2400	310
Magnesium	71,162.80	–	–	–	–	–
Manganese	1038.72	–	–	–	–	–
Molybdenum	356.17	–	–	–	–	–
Nickel	219.09	310	2060	1500	6900	1500
Platinum	0.84	–	–	–	–	–
Potassium	363,040.57	–	–	–	–	–
Selenium	6.62	–	–	–	–	–
Silver	0.28	150	3340	400	2500	950
Sodium	1,124,646.59	–	–	–	–	–
Thallium	0.36	–	–	–	–	–
Thorium	0.20	–	–	–	–	–
Uranium	0.71	–	–	–	–	–
Vanadium	7.68	–	–	–	–	–
Zinc	397.39	1140	8200	8200	5300	8360

^*a*^ Permit limits from Louisville/Jefferson County Metropolitan Sewer District (2022)

### Quality control samples

The U.S. EPA [40] wastewater sampling procedure recommends field rinse blank samples, especially in cases of possible low-level contamination. One field rinse blank was collected during field sample collection (Table S5). Despite the use of high-purity water, 24 of 26 metals were detected at quantifiable levels, indicating practical challenges in this type of investigation. There was a high degree of overlap; however, the two metals below the limit of quantification (chromium and thallium) were generally not detected across the wastewater sampling locations. The source of contamination (sampling equipment or water) was not investigated and should be considered for future studies.

## Discussion

Wastewater surveillance can inform health equity discussions [7, 41], especially in conditions where toxicity data are limited or unavailable at certain places and time periods. Wastewater may provide an additional, complementary environmental matrix to identify which residents are exposed to environmental toxicants, guide remediation, prioritize federal and state regulatory requirements, and, as needed, support emergency surveillance. As it is uncommon to measure blood and/or urine levels of many of the metals assessed in our study, apart from childhood blood Pb levels, wastewater surveillance provides an additional opportunity to evaluate community-level concentrations. Although there was some variability in the 3-week intra-site assessment at site E23, these results indicate a high likelihood that the nearly 38,000 residents of this sewershed have higher Pb exposure than residents in other areas. The gradients of several metals observed in this study suggest that wastewater may provide additional complementary data for identifying smaller neighborhood- or multi-neighborhood areas of exposure concern within an urban area with known environmental sources of pollution.

The mean concentrations of the metals analyzed in this study were higher than those in tap water samples. Accordingly, the elevated wastewater concentrations likely reflect contributions from environmental sources, excretion by humans associated with environmental exposure (e.g., dietary or inhalation exposure among residents), or industrial dumping into the sewer system. Though the latter is unlikely to occur in several sewersheds without proximity to known environmental sources of pollution (e.g., E17; Fig. 1). There is also a role for the American Society for Testing and Materials standards in assessing wastewater sampling for metals as part of large-scale natural disasters or chemical post-incident emergency response or impact surveillance. One weakness of wastewater-based surveillance is the inability to differentiate occupational exposures from other exposures and from excreted substances.

Ba in the environment can result from burning coal [42–44]. Two sites had especially high Ba levels (sites E8 and E28), both having a combined sewer system. Site E8 is across the Ohio River from the Ohio Falls Power Plant, and Site E28 is near the Cane Run Power Plant. Both plants burned coal in the recent past and have large on-site coal ash storage. Thus, the high concentrations could reflect particulates settling on roadways and being deposited in sewers with rainwater runoff in a combined sewer system, or inhalation exposure with excreted metals (or their metabolites) in urine. The discovery of high Ba levels in the dedicated sanitary sewer systems throughout the county indicates that the metal is likely to originate from human exposure and excretion. However, industrial discharge into the wastewater system cannot be completely excluded at this time. Follow-up sampling at sites surrounding E8 and E28 will be required to draw firm conclusions regarding exposures.

The west side of Louisville has two power plants along the Ohio River, the Mill Creek Generating Station and the Cane Run Generating Station. The Mill Creek Generating Station is the largest coal-fired power plant operated by Louisville Gas & Electric company [45]. The Cane Run Plant (near Site E28) converted from burning coal to burning natural gas in 2015, thereby reducing environmental exposure to contaminants [46]. However, an ash waste pile and a slurry pond remain at the site, and residents living within 10 miles of the site exhibit evidence of heavy metal contamination from coal ash [47]. Both Ba and As were detected in toenails of the children studied by this group [48]; K.M. Zierold, personal communication], evidence of body burden from exposure to these metals. Although As in food may contribute to its presence in toenails, coal ash contributes to environmental pollution by As and other metals. Thus, airborne coal ash is likely the major contributor to the body burden of heavy metals [49] in this area of our study.

### Future research

Future research should address several priorities for community‑wide exposome surveillance using wastewater. Although comparability of 24‑hour composite and grab wastewater samples has been studied for WBE pathogen targets [50], it has not been systematically examined for metals. In addition, research is needed to determine whether influent wastewater and settled solids provide comparable results. Finer spatial and temporal sampling (across different times of day and on different days) is needed to gain a more complete picture of spatial heterogeneity in metal background levels and population exposure sensitivity. Time‑series sampling may also better reveal exposure dynamics in the study area and allow further analysis that integrates population and land‑use covariates, including seasonal or event‑driven repeat sampling, to examine metal fluctuations alongside rainfall and industrial discharge cycles.

Larger sample sizes are needed to statistically determine areas of concern, to explicitly compare tap water and wastewater concentrations, to evaluate correlations among the different metals tested (i.e., whether sites elevated in one metal are also more likely to be elevated in others), and to assess factors—such as socio-economic status and land use—that may correlate with increased metals in wastewater. Because our study included both combined and non‑combined sewer systems, metals present in the combined systems may reflect runoff from rain on streets and soil as well as effluent from households and businesses; this requires further investigation as it represents other avenues of exposure.

Future research should also incorporate metal concentration analyses of human biospecimens (from research cohorts) paired with sewershed‑scale wastewater concentrations. Results from the field duplicate and rinse blank underscore the importance of incorporating rigorous quality control samples to evaluate potential contamination during sampling and handling activities. Further studies should follow or supplement the U.S. EPA [40] wastewater sampling procedure to strengthen data reliability. Additionally, exposome case studies that include metals in wastewater following large‑scale natural disasters or chemical incidents are needed to support emergency response efforts, as well as epidemiological surveys assessing population-level exposure to metals, travel to other regions, and water and food consumption behaviors.

### Limitations

Metals are challenging to evaluate for exposure because they occur in the environment (including in soils, water, food, and cigarette smoke), and metal concentrations in wastewater reflect multiple potential environmental sources, including industrial discharges, runoff, and aging infrastructure. These confounding factors—along with dilution effects and other environmental parameters—can interfere with conclusions and currently limit the ability to use wastewater data to accurately infer human exposure or contamination. This study is limited by a small sample size and a single cross-section, without the use of time-series sampling. Most sites relied on a single grab sample, with composite samples collected at only one location. Although the samples provide good representation across the county at a specific point in time, composite samples from all sites would yield a more detailed assessment. Contamination detected in the field blank also indicates that the sample collection protocols warrant further investigation, despite adherence to standard WBE sampling protocols [15].

The proximity to a Superfund site and other environmental pollution sources may not necessarily pose an exposure risk, depending on the type of contamination present at these facilities and whether the health-risk conceptual site model indicates an exposure pathway to nearby residents. Additionally, information on household plumbing materials, including pipes, faucet fixtures, and soldering was unavailable, all of which may contribute metals to wastewater. The study does not attempt to estimate human metal intake or associated health risks; therefore, quantitative statements about human exposure cannot be made without additional data sources, such as biospecimens from clinical research participants or individual-level case data.

## Conclusion

This initial study provides preliminary evidence supporting the use of wastewater-based surveillance for community-scale exposome assessment by revealing spatial variability in metal levels across a single county, highlighting the need for further research. The mean values for wastewater metal concentrations analyzed in our study were higher than the levels in tap water samples; thus, the increases observed in the wastewater likely reflect contributions from environmental sources, excretion from humans reflecting body burden from exposure (e.g., either dietary or inhalation exposure to residents), or industrial input into the sewer system. That metals in wastewater can indirectly capture soil, water, air quality, and human exposure within an area, as shown by a single sample within their corresponding sewersheds, provides an innovative expansion of exposome assessment to the community level. Surveillance of metals in wastewater may provide an opportunity to examine integrated toxicology to complement other toxicology data, identify where people live, and determine which toxicants warrant further research.

## Supplementary Information

Below is the link to the electronic supplementary material.

Supplementary Material 1

## Data Availability

Data generated in this study can be found in the article and its supplementary files.
