# Supplementary material for: Wastewater-based surveillance for community exposome surveillance, Louisville, Kentucky
Source: Discov Water. 2026 Mar 12;6(1):57. doi: 10.1007/s43832-026-00376-5 (PMC13095899; doi:10.1007/s43832-026-00376-5)
Supplement: Supplementary file 1 — Supplementary Material 1 [file 43832_2026_376_MOESM1_ESM.docx]

**Supplementary Materials**

**Wastewater-based surveillance for community exposome surveillance, Louisville, Kentucky**

Lu Cai^1,2^, Rochelle H. Holm^3^*, Donald J. Biddle^4,5^, Charlie H. Zhang^2,4^, Daymond Talley^6^, Ted Smith^2,3#^, and J. Christopher States^2,7#^*

^1^Department of Pediatrics, Pediatrics Research Institute, University of Louisville, Louisville, KY, USA

^2^Center for Integrative Environmental Health Sciences, University of Louisville, Louisville, KY, USA

^3^Center for Healthy Air Water and Soil, Christina Lee Brown Envirome Institute, University of Louisville, Louisville, KY, USA

^4^Department of Geographic and Environmental Sciences, University of Louisville, Louisville, KY, USA

^5^Center for Geographic Information Sciences, University of Louisville, Louisville, KY, USA

^6^Louisville/Jefferson County Metropolitan Sewer District, Morris Forman Water Quality Treatment Center, Louisville, KY, USA

^7^Department of Pharmacology and Toxicology, University of Louisville, Louisville, KY, USA

^#^Joint senior authors

*Joint corresponding authors:

Rochelle H. Holm, Center for Healthy Air Water and Soil, Christina Lee Brown Envirome Institute, University of Louisville, 302 E. Muhammad Ali Blvd., Louisville, KY 40202, USA; [rochelle.holm@louisville.edu](mailto:rochelle.holm@louisville.edu)

J. Christopher States, Center for Integrative Environmental Health Sciences, Department of Pharmacology and Toxicology, University of Louisville, 505 S. Hancock St. Rm 304, Louisville, KY 40202, USA; [jcstates@louisville.edu](mailto:jcstates@louisville.edu)

**Table of Contents**

[Table S1. Metals studied by Inductively Coupled Plasma Quadrupole Mass Spectrometry, detection limit, and standard curve levels. 3](#_Toc221098680)

[Table S2. Wastewater metal concentrations by site (grab samples) (N = 25). 4](#_Toc221098681)

[Table S3. Wastewater metal concentrations by date (composite samples) at site E23 (N=9). 6](#_Toc221098682)

[Table S4. Tap water metal concentration by site (N=5). 7](#_Toc221098683)

[Table S5. Metal concentrations in the rinsate blank field sample. 8](#_Toc221098684)

# Table S1. Metals studied by Inductively Coupled Plasma Quadrupole Mass Spectrometry, detection limit, and standard curve levels.

| **Metal** | **Detection limit (ng/mL)** | **Standard curve level** |
| --- | --- | --- |
| Ag | 0.002 | 0, 1, 2, 5, 10, 50 ng |
| AI | 0.357 | 0, 1, 2, 5, 10, 50 ng |
| As | 0.007 | 0, 1, 2, 5, 10, 50 ng |
| Ba | 0.008 | 0, 1, 2, 5, 10, 50 ng |
| Be | 0 | 0, 1, 2, 5, 10, 50 ng |
| Ca | 21.82 | 0, 100, 200, 500, 1000, 5000 ng |
| Cd | 0 | 0, 1, 2, 5, 10, 50 ng |
| Co | 0.003 | 0, 1, 2, 5, 10, 50 ng |
| Cr | 0.038 | 0, 1, 2, 5, 10, 50 ng |
| Cu | 0.009 | 0, 1, 2, 5, 10, 50 ng |
| Fe | 0.1 | 0, 100, 200, 500, 1000, 5000 ng |
| K | 8.31 | 0, 100, 200, 500, 1000, 5000 ng |
| Mg | 0.064 | 0, 100, 200, 500, 1000, 5000 ng |
| Mn | 0.043 | 0, 1, 2, 5, 10, 50 ng |
| Mo | 0.005 | 0, 1, 2, 5, 10, 50 ng |
| Na | 4.23 | 0, 100, 200, 500, 1000, 5000 ng |
| Ni | 0.031 | 0, 1, 2, 5, 10, 50 ng |
| Pb | 0 | 0, 1, 2, 5, 10, 50 ng |
| Pt | 0 | 0, 1, 2, 5, 10, 50 ng |
| Sb | 0.003 | 0, 1, 2, 5, 10, 50 ng |
| Se | 0.321 | 0, 1, 2, 5, 10, 50 ng |
| Th | 0.001 | 0, 1, 2, 5, 10, 50 ng |
| TI | 0.001 | 0, 1, 2, 5, 10, 50 ng |
| U | 0.001 | 0, 1, 2, 5, 10, 50 ng |
| V | 0.006 | 0, 1, 2, 5, 10, 50 ng |
| Zn | 0.136 | 0, 1, 2, 5, 10, 50 ng |

# Table S2. Wastewater metal concentrations by site (grab samples) (N = 25).

|  | **Ag** | **Al** | **As** | **Ba** | **Be** | **Ca** | **Cd** | **Co** | **Cr** | **Cu** | **Fe** | **K** | **Mg** |
| --- | --- | --- | --- | --- | --- | --- | --- | --- | --- | --- | --- | --- | --- |
| **Sample ID** | **ng/mL** | **ng/mL** | **ng/mL** | **ng/mL** | **ng/mL** | **ng/mL** | **ng/mL** | **ng/mL** | **ng/mL** | **ng/mL** | **ng/mL** | **ng/mL** | **ng/mL** |
| E1 | 0.040 | 8580 | 1.093 | 53.147 | 0.000 | 27645 | 0.107 | 0.440 | 0.280 | 72.080 | 392.52 | 3967 | 11896 |
| E2 | 0.040 | 142 | 0.773 | 31.293 | 0.093 | 54968 | 0.027 | 0.400 | 0.560 | 8.800 | 498.03 | 10835 | 13820 |
| E3 | 0.040 | 53 | 1.760 | 40.453 | 0.000 | 63087 | 0.013 | 0.333 | 0.280 | 7.173 | 665.27 | 17957 | 22103 |
| E4 | 0.280 | 151 | 1.027 | 60.613 | 0.187 | 48311 | 0.027 | 0.573 | 0.380 | 13.053 | 505.32 | 12648 | 16581 |
| E5 | 0.227 | 80 | 1.027 | 37.467 | 0.280 | 53295 | 0.173 | 7.493 | 1.680 | 15.667 | 244.43 | 8199 | 18402 |
| E7 | 0.027 | 110 | 0.973 | 27.613 | 0.093 | 37148 | 0.080 | 0.400 | 0.360 | 13.093 | 287.15 | 11495 | 12220 |
| E8 | 0.067 | 212 | 1.040 | 399.413 | 0.000 | 41114 | 0.080 | 0.573 | 1.820 | 12.907 | 1154.79 | 14983 | 13506 |
| E9 | 0.040 | 20355 | 1.200 | 54.147 | 1.680 | 63572 | 0.067 | 0.480 | 0.973 | 8.293 | 597.85 | 230955 | 16127 |
| E10 | 0.067 | 56 | 0.653 | 48.840 | 0.280 | 72859 | 0.040 | 0.187 | 0.320 | 10.947 | 171.97 | 5203 | 14174 |
| E11 | 0.107 | 120 | 0.453 | 42.987 | 0.000 | 66355 | 0.107 | 0.240 |  | 11.720 | 262.61 | 5075 | 12354 |
| E12 | 0.040 | 19104 | 1.493 | 60.453 | 2.160 | 67386 | 0.093 | 0.467 | 1.400 | 34.187 | 866.28 | 261422 | 21387 |
| E13 | 0.053 | 93 | 0.440 | 41.480 | 0.093 | 71186 | 0.080 | 0.280 | 5.787 | 10.400 | 598.20 | 6047 | 18655 |
| E14 | 0.160 | 138 | 0.333 | 39.507 | 0.000 | 70766 | 0.120 | 0.427 | 1.800 | 29.587 | 297.44 | 9497 | 13500 |
| E15 | 0.080 | 573 | 0.693 | 43.547 | 0.093 | 46049 | 0.040 | 0.653 | 1.520 | 16.413 | 509.61 | 20782 | 20317 |
| E16 | 0.020 | 286 | 0.320 | 42.213 | 0.093 | 30519 | 0.027 | 0.533 |  | 3.453 | 99.39 | 20650 | 13063 |
| E17 | 0.253 | 144 | 0.667 | 40.707 | 0.093 | 78044 | 0.067 | 0.227 | 0.040 | 13.853 | 173.47 | 9646 | 23073 |
| E18 | 0.120 | 192 | 0.627 | 44.480 | 0.000 | 67560 | 0.480 | 0.573 | 0.240 | 32.667 | 509.69 | 9305 | 19137 |
| E19 | 0.067 | 121 | 0.693 | 36.613 | 0.093 | 66923 | 0.027 | 0.267 | 0.520 | 12.453 | 226.56 | 11526 | 26593 |
| E20 | 0.093 | 127 | 0.373 | 39.680 | 0.187 | 54919 | 0.053 | 0.267 | 0.040 | 16.853 | 195.85 | 10668 | 24562 |
| E22 | 0.080 | 111 | 0.707 | 45.427 | 0.093 | 58811 | 0.027 | 0.320 | 0.360 | 14.747 | 271.15 | 11241 | 26226 |
| E23 | 0.080 | 201 | 0.840 | 29.680 | 0.093 | 50687 | 0.067 | 0.373 | 1.600 | 23.627 | 657.33 | 16272 | 18862 |
| E24 | 0.133 | 127 | 0.733 | 33.333 | 0.373 | 42384 | 0.053 | 0.347 | 5.200 | 23.947 | 421.32 | 13801 | 15209 |
| E25 | 0.027 | 42 | 0.493 | 39.147 | 0.000 | 70539 | 0.040 | 0.333 |  | 11.880 | 480.37 | 16802 | 18333 |
| E26 | 0.040 | 17394 | 1.560 | 29.347 | 1.880 | 45913 | 0.053 | 0.573 | 6.880 | 9.227 | 496.04 | 363041 | 16422 |
| E28 | 0.040 | 152 | 0.720 | 199.133 | 0.280 | 3410126 | 0.173 | 2.067 |  | 19.947 | 151.56 | 22609 | 71163 |

|  | **Mn** | **Mo** | **Na** | **Ni** | **Pb** | **Pt** | **Sb** | **Se** | **Th** | **Tl** | **U** | **V** | **Zn** |
| --- | --- | --- | --- | --- | --- | --- | --- | --- | --- | --- | --- | --- | --- |
| **Sample ID** | **ng/mL** | **ng/mL** | **ng/mL** | **ng/mL** | **ng/mL** | **ng/mL** | **ng/mL** | **ng/mL** | **ng/mL** | **ng/mL** | **ng/mL** | **ng/mL** | **ng/mL** |
| E1 | 23.99 | 356.173 | 281538 | 87.387 | 0.707 | 0.120 | 0.187 | 5.000 | 0.133 | 0.000 | 0.133 | 0.507 | 15.453 |
| E2 | 116.47 | 1.667 | 29330 | 4.027 | 1.613 | 0.133 | 0.253 | 2.640 | 0.107 | 0.000 | 0.187 | 0.427 | 38.053 |
| E3 | 201.25 | 1.387 | 49938 | 6.547 | 0.787 | 0.133 | 0.187 | 3.960 | 0.040 |  | 0.240 | 0.213 | 26.347 |
| E4 | 81.57 | 1.160 | 81944 | 6.107 | 1.427 | 0.133 | 0.320 | 4.860 | 0.027 |  | 0.307 | 0.440 | 36.907 |
| E5 | 79.04 | 3.827 | 48540 | 219.093 | 1.027 | 0.120 | 0.533 | 1.760 | 0.200 | 0.360 | 0.347 | 0.547 | 35.173 |
| E7 | 40.85 | 0.813 | 42983 | 1.640 | 1.867 | 0.133 | 0.333 | 6.180 | 0.000 | 0.000 | 0.453 | 0.427 | 28.213 |
| E8 | 233.61 | 1.493 | 46298 | 2.787 | 4.227 | 0.120 | 0.640 | 4.720 | 0.000 |  | 0.493 | 7.680 | 46.013 |
| E9 | 158.79 | 2.267 | 538474 | 1.693 | 0.813 | 0.387 | 0.333 | 4.420 | 0.027 | 0.020 | 0.467 | 1.573 | 78.413 |
| E10 | 82.40 | 0.320 | 39067 | 1.240 | 0.773 | 0.107 | 0.133 | 6.620 | 0.000 |  | 0.453 | 0.280 | 33.547 |
| E11 | 73.99 | 0.360 | 39920 | 1.467 | 2.680 | 0.120 | 0.147 | 2.200 | 0.000 |  | 0.413 | 0.307 | 43.227 |
| E12 | 178.15 | 2.173 | 519013 | 2.787 | 1.253 | 0.293 | 0.507 | 1.320 | 0.013 | 0.000 | 0.493 | 1.880 | 54.600 |
| E13 | 53.69 | 0.613 | 56266 | 2.493 | 1.173 | 0.107 | 0.187 | 4.400 | 0.067 |  | 0.440 | 0.493 | 397.387 |
| E14 | 49.73 | 0.747 | 45989 | 2.320 | 2.027 | 0.107 | 0.200 | 2.200 | 0.040 | 0.060 | 0.467 | 0.440 | 79.733 |
| E15 | 27.91 | 45.787 | 83557 | 4.707 | 3.240 | 0.133 | 0.787 | 2.053 | 0.013 | 0.000 | 0.413 | 0.493 | 115.667 |
| E16 | 18.39 | 79.253 | 78686 | 4.320 | 0.133 | 0.133 | 1.173 | 1.320 |  |  | 0.347 | 0.267 | 57.933 |
| E17 | 22.52 | 1.467 | 49306 | 1.147 | 0.373 | 0.093 | 0.320 | 3.080 | 0.000 |  | 0.467 | 0.347 | 72.520 |
| E18 | 86.19 | 2.187 | 55692 | 3.160 | 2.587 | 0.093 | 0.240 | 2.200 | 0.000 | 0.000 | 0.493 | 0.613 | 70.893 |
| E19 | 22.92 | 1.147 | 56050 | 1.400 | 0.400 | 0.093 | 0.333 | 2.200 | 0.000 | 0.000 | 0.547 | 0.307 | 52.040 |
| E20 | 30.60 | 0.653 | 46599 | 1.907 | 0.880 | 0.120 | 0.373 | 3.960 | 0.000 | 0.000 | 0.600 | 0.320 | 64.333 |
| E22 | 68.13 | 1.000 | 43767 | 1.720 | 1.200 | 0.093 | 0.307 | 3.520 | 0.000 | 0.000 | 0.493 | 0.427 | 60.560 |
| E23 | 72.92 | 1.280 | 48357 | 2.733 | 8.800 | 0.080 | 0.520 | 5.280 | 0.027 |  | 0.267 | 0.453 | 75.800 |
| E24 | 84.65 | 0.813 | 56362 | 3.347 | 2.093 | 0.093 | 0.267 | 3.980 | 0.000 |  | 0.293 | 0.267 | 56.613 |
| E25 | 80.11 | 1.000 | 62629 | 4.800 | 0.587 | 0.093 | 0.213 | 3.520 | 0.000 | 0.000 | 0.427 | 0.360 | 27.120 |
| E26 | 128.07 | 2.440 | 630457 | 2.973 | 1.653 | 0.147 | 0.320 | 3.080 | 0.000 | 0.000 | 0.467 | 2.960 | 40.253 |
| E28 | 1038.72 | 8.040 | 1124647 | 7.613 | 1.053 | 0.840 | 0.960 | 3.227 | 0.000 | 0.013 | 0.707 | 0.960 | 66.133 |

# Table S3. Wastewater metal concentrations by date (composite samples) at site E23 (N=9).

| **Date:** | **1/9/2024** | **1/10/2024** | **1/11/2024** | **1/16/2024** | **1/17/2024** | **1/18/2024** | **1/23/2024** | **1/24/2024** | **1/25/2024** |  |  |  |
| --- | --- | --- | --- | --- | --- | --- | --- | --- | --- | --- | --- | --- |
| **Sample ID:** | **WT1** | **WT2** | **WT3** | **WR4** | **WT5** | **WT6** | **WT7** | **WT8** | **WT9** | mean | STDEV | median |
| **Metal** | Metal concentration (ng/mL) | | | | | | | | | | | |
| Ag | 0.32 | 0.09 | 0.12 | 0.60 | 0.16 | 0.05 | 0.16 | 0.08 | 0.07 | 0.18 | 0.18 | 0.12 |
| Al | 1052 | 390 | 206 | 240 | 299 | 194 | 817 | 215 | 571 | 443 | 309 | 299 |
| As | 1.09 | 1.01 | 0.23 | 0.36 | 0.53 | 0.48 | 0.76 | 0.19 | 0.89 | 0.62 | 0.34 | 0.53 |
| Ba | 76.16 | 24.80 | 28.88 | 27.28 | 49.76 | 33.60 | 50.60 | 30.87 | 37.69 | 39.96 | 16.44 | 33.60 |
| Be | 0.20 | 0.00 | 0.00 | 0.10 | 0.00 | nd | nd | 0.10 | nd | nd | nd | nd |
| Ca | 59,508 | 36,816 | 38,203 | 43,304 | 49,839 | 48,563 | 48,013 | 46,288 | 46,512 | 46,339 | 6,704 | 46,512 |
| Cd | 0.37 | 0.09 | 0.08 | 0.09 | 0.19 | 0.15 | 0.37 | 0.11 | 0.16 | 0.18 | 0.12 | 0.15 |
| Co | 1.04 | 0.41 | 0.37 | 0.43 | 0.69 | 0.55 | 0.96 | 0.45 | 0.52 | 0.60 | 0.24 | 0.52 |
| Cr | 8.04 | 2.49 | 2.29 | 2.72 | 9.44 | 5.60 | 5.27 | 2.13 | 2.49 | 4.50 | 2.75 | 2.72 |
| Cu | 61.43 | 16.23 | 20.51 | 35.47 | 32.33 | 21.84 | 52.11 | 26.09 | 17.64 | 31.52 | 15.83 | 26.09 |
| Fe | 2529 | 606 | 476 | 310 | 497 | 596 | 1460 | 526 | 783 | 865 | 705 | 596 |
| K | 101,178 | 24,229 | 19,021 | 12,385 | 19,892 | 23,823 | 58,388 | 21,046 | 31,337 | 34,589 | 28,216 | 23,823 |
| Mg | 15,834 | 9,155 | 10,589 | 11,765 | 16,034 | 15,834 | 14,878 | 10,185 | 12,548 | 12,980 | 2,717 | 12,548 |
| Mn | 78.35 | 33.68 | 41.97 | 32.32 | 52.69 | 54.63 | 89.32 | 42.39 | 45.80 | 52.35 | 19.52 | 45.80 |
| Mo | 4.32 | 1.95 | 1.44 | 2.45 | 3.56 | 9.43 | 4.56 | 1.40 | 1.44 | 3.39 | 2.58 | 2.45 |
| Na | 51,359 | 25,651 | 40,398 | 38,650 | 58,536 | 44,611 | 51,114 | 33,826 | 71,211 | 46,151 | 13,679 | 44,611 |
| Ni | 9.21 | 1.65 | 1.45 | 1.60 | 5.23 | 2.95 | 5.79 | 1.97 | 1.29 | 3.46 | 2.73 | 1.97 |
| Pb | 11.51 | 1.52 | 2.27 | 1.56 | 2.85 | 1.89 | 7.15 | 2.21 | 2.04 | 3.67 | 3.41 | 2.21 |
| Pt | 0.04 | 0.00 | 0.00 | 0.00 | 0.00 | 0.01 | 0.04 | 0.00 | 0.00 | 0.01 | 0.02 | 0.00 |
| Sb | 1.29 | 0.57 | 0.41 | 0.93 | 2.05 | 0.53 | 1.28 | 0.57 | 0.59 | 0.92 | 0.54 | 0.59 |
| Se | 3.47 | 2.11 | 2.11 | 0.76 | 1.96 | 1.58 | 2.50 | 3.62 | 1.67 | 2.20 | 0.90 | 2.11 |
| Th | 0.64 | 0.25 | 0.13 | 0.12 | 0.09 | 0.08 | 0.12 | 0.04 | 0.12 | 0.18 | 0.18 | 0.12 |
| Tl | 0.05 | 0.00 | 0.00 | 0.00 | 0.01 | 0.00 | 0.04 | 0.00 | 0.01 | 0.01 | 0.02 | 0.00 |
| U | 0.49 | 0.25 | 0.33 | 0.24 | 0.32 | 0.31 | 0.47 | 0.21 | 0.29 | 0.32 | 0.10 | 0.31 |
| V | 2.31 | 1.01 | 0.51 | 0.48 | 0.68 | 0.53 | 1.56 | 0.41 | 1.20 | 0.97 | 0.64 | 0.68 |
| Zn | 279.21 | 43.76 | 69.89 | 64.15 | 100.11 | 78.89 | 186.93 | 97.16 | 74.00 | 110.46 | 75.15 | 78.89 |

# Table S4. Tap water metal concentration by site (N=5).

| **Sample concentration (ng/mL)** | | | | | |
| --- | --- | --- | --- | --- | --- |
| **Metal/Sample ID** | **C1** | **C2** | **C4** | **C5** | **C6** |
| Ag | 0.16 | 0 | 0.05 | 0.05 | 0.24 |
| Al | 66 | 63 | 145.16 | 195.52 | 191.12 |
| As | 1.12 | 0.88 | 0.29 | 0.09 | 0.17 |
| Ba | 19 | 20 | 27.68 | 26.43 | 26.84 |
| Be | 0 | 0 | 0.21 | 0.43 | 0.16 |
| Ca | 30648 | 32531 | 33101.36 | 33279.45 | 33558.48 |
| Cd | 0 | 0.04 | 0.04 | 0.06 | 0.01 |
| Co | 0.08 | 0.04 | 0.19 | 0.23 | 0.23 |
| Cr | 3.32 | 2.44 | 1.63 | 1.83 | 3.00 |
| Cu | 26 | 27 | 10.88 | 23.55 | 7.63 |
| Fe | 97 | 36 | 121.36 | 123.61 | 160.20 |
| K | 3060 | 3291 | 3317.31 | 3395.76 | 3458.20 |
| Mg | 14016 | 14477 | 13989.85 | 14133.12 | 13969.49 |
| Mn | 2.32 | 2.12 | 3.12 | 3.28 | 3.85 |
| Mo | 2.68 | 2.32 | 2.03 | 1.87 | 1.92 |
| Na | 28302 | 29114 | 38307.16 | 38396.84 | 37870.05 |
| Ni | 1.2 | 0.8 | 1.57 | 1.33 | 1.55 |
| Pb | 1.36 | 1.6 | 0.29 | 0.52 | 0.29 |
| Pt | 0 | 0 | 0 | 0 | 0 |
| Sb | 0.32 | 0.28 | 0.23 | 0.17 | 0.21 |
| Se | 0.96 | 0 | 3.78 | 2.01 | 1.02 |
| Th | 0 | 0 | 0.20 | 0.12 | 0.09 |
| Tl | 0 | 0 | 0.00 | 0.00 | 0.00 |
| U | 0.28 | 0.32 | 0.28 | 0.28 | 0.28 |
| V | 0.92 | 0.84 | 0.47 | 0.49 | 0.53 |
| Zn | 27 | 6.68 | 31.08 | 32.63 | 27.32 |
|  | | | | | |

# Table S5. Metal concentrations in the rinsate blank field sample.

| **Metal** | **Concentration (ng/mL)** |
| --- | --- |
| Ag | 0.120 |
| Al | 235 |
| As | 0.467 |
| Ba | 40.347 |
| Be | 0.093 |
| Ca | 46103 |
| Cd | 0.053 |
| Co | 0.427 |
| Cr | Below quantification |
| Cu | 6.573 |
| Fe | 108.92 |
| K | 16837 |
| Mg | 16485 |
| Mn | 19.65 |
| Mo | 52.600 |
| Na | 68647 |
| Ni | 3.280 |
| Pb | 0.253 |
| Pt | 0.107 |
| Sb | 0.933 |
| Se | 2.347 |
| Th | 0.000 |
| Tl | Below quantification |
| U | 0.440 |
| V | 0.320 |
| Zn | 60.253 |
